# Pharmacogenetics of Direct Oral Anticoagulants: A Systematic Review

**DOI:** 10.3390/jpm11010037

**Published:** 2021-01-11

**Authors:** Johanna Raymond, Laurent Imbert, Thibault Cousin, Thomas Duflot, Rémi Varin, Julien Wils, Fabien Lamoureux

**Affiliations:** 1 Pharmacy Department, Rouen University Hospital, 76031 Rouen, France; johanna.raymond@chu-rouen.fr (J.R.); thibault_cousin@hotmail.fr (T.C.); remi.varin@chu-rouen.fr (R.V.); 2 Laboratory of Pharmacology, Toxicology and Pharmacogenetic, Pharmacology Department, Rouen University Hospital, 76031 Rouen, France; laurent.imbert@chu-rouen.fr (L.I.); julien.wils@chu-rouen.fr (J.W.); 3 Department of Pharmacology, Normandie University, UNIROUEN, INSERM U1096, CHU Rouen, F-76000 Rouen, France; thomas.duflot@chu-rouen.fr

**Keywords:** direct oral anticoagulants, pharmacogenetics, adverse drug reactions, clinical implementation

## Abstract

Dabigatran, rivaroxaban, apixaban, edoxaban, and betrixaban are direct oral anticoagulants (DOACs). Their inter-individual variability in pharmacodynamics and pharmacokinetics (transport and metabolism) is high, and could result from genetic polymorphisms. As recommended by the French Network of Pharmacogenetics (RNPGx), the management of some treatments in cardiovascular diseases (as antiplatelet agents, oral vitamin K antagonists, and statins) can rely on genetic testing in order to improve healthcare by reducing therapeutic resistance or toxicity. This paper is a review of association studies between single nucleotide polymorphisms (SNPs) and systemic exposure variation of DOACs. Most of the results presented here have a lot to do with some SNPs of CES1 (rs2244613, rs8192935, and rs71647871) and ABCB1 (rs1128503, rs2032582, rs1045642, and rs4148738) genes, and dabigatran, rivaroxaban, and apixaban. Regarding edoxaban and betrixaban, as well as SNPs in the *CYP3A4* and *CYP3A5* genes, literature is scarce, and further studies are needed.

## 1. Introduction

Dabigatran, rivaroxaban, apixaban, edoxaban, and betrixaban are direct oral anticoagulants (DOACs). Their mechanism of action is based on the direct inhibition of coagulation factors: either thrombin (factor IIa) for dabigatran, or Stuart factor (Xa) for rivaroxaban, apixaban, edoxaban, and betrixaban. DOACs are alternative treatments to oral anti-vitamin K anticoagulants (AVK: fluindione, warfarin, and acenocoumarol). However, the inter-individual variability of these treatments is significant, and can lead to hemorrhagic or thromboembolic events. This variability could be related to polymorphisms of genes coding for proteins responsible for the activation, transport, or metabolism of DOACs, such as *CES1*, *ABCB1*, *CYP3A4*, and *CYP3A5* (Table 1). Their pharmacokinetic and pharmacodynamic variability is also impacted by drug interactions when CYP450 or P-glycoprotein inducers or inhibitors are co-administered. DOACs are not subject to pharmacogenetic testing in clinical practice, unlike other cardiovascular drugs (antiplatelet agents, anti-vitamin K, and statins), for which such testing is recommended [1].

## 2. Materials and Methods

A literature review was conducted using PubMed in order to identify studies evaluating the impact of CYP genetic polymorphisms on DOAC exposure, taking into account adverse events. The terms “DABIGATRAN”, “RIVAROXABAN”, “APIXABAN”, “EDOXABAN”, and “BETRIXABAN” have been crossed with “PHARMACOGENETICS”, “PHARMACOGENOMICS”, “POLYMORPHISM”, “CYP3A4”, “CYP3A5”, or “ABCB1” and “BLEEDING”, “HEMORRHAGE”, or “THROMBOEMBOLIC EVENTS”. “DABIGATRAN” and “EDOXABAN” have been also crossed with “CES”.

## 3. Results

### 3.1. Dabigatran

#### 3.1.1. Pharmacodynamics and Pharmacokinetics

Dabigatran is administered as a prodrug dabigatran etexilate. Its bioavailability is 7% [7]. It is a P-glycoprotein substrate, which implies drug interactions with potent P-glycoprotein inducers (rifampin, St. John’s wort, carbamazepine, phenytoin, etc.) and P-glycoprotein inhibitors (systemic ketoconazole, itraconazole, ritonavir, cyclosporine, clarithromycin, dronedarone, amiodarone, quinidine, verapamil, ticagrelor) [3,7]. Dabigatran etexilate is activated by intestinal (CES2 isoform) and hepatocyte (CES1 isoform) carboxylesterases (CES) to form short-lived metabolites, BIBR 951 and BIBR 1087. Non-enzymatic hydrolysis also converts the prodrug to BIBR 1087. The intermediate metabolites are in turn hydrolyzed by CES1 in hepatocytes to yield active dabigatran [8,9]. The plasma protein binding of dabigatran is 35% [10]. Dabigatran is metabolized to a small extent (< 10%) by the UDP-glucuronyltransferase (UGTs) isoforms 1A9, 2B7, and 2B15, leading to the formation of four active metabolites [9]. Dabigatran is not metabolized by CYP450 and does not induce or inhibit CYP450, except at supra-therapeutic concentrations (in vitro at 100 μM: inhibition of CYP3A4 and CYP2E1) [7,10]. Dabigatran and its metabolites are eliminated mainly via the urinary route (80–90%). It has a relatively long plasma elimination half-life of 12–17 h [7].

#### 3.1.2. Pharmacogenetics

##### Genetic Polymorphism of *CES1*

The *CES1* and *CES2* genes are located on chromosome 16, and contain 14 and 12 exons respectively. In humans, the CES1 protein is the most hepatically active isoform, with approximately 90% of the activity [11]; 2000 polymorphisms have been described for *CES1* [12]. The single nucleotide polymorphisms (SNPs) rs2244613 (C > A), rs8192935 (T > C), and rs71647871 (G > A) [13] have been associated with pharmacokinetic variations of dabigatran [11,14,15,16,17] (Table 2). The first two SNPs are in incomplete linkage disequilibrium (r^2^ = 0.45) [14], and their impact on the expression or activity of CES1 has not been clearly established, unlike rs71647871, which induces a loss of CES1 function by substitution of one of the three glycines at the active site by a glutamate [12]. Overall, these three SNPs lead to a decrease in systemic exposure to dabigatran, reducing the risk of hemorrhage, without thromboembolic events being associated [12,14].

##### Genetic Polymorphism of *ABCB1*

The *ABCB1* gene is located on chromosome 7 and contains 29 exons (4872 bp) [28]. In 2009, 1279 SNPs, including 22 silent mutations, 41 nonsense mutations and one in the start codon, were known [29]. The most common polymorphisms are rs1128503 (1236 C > T), rs2032582 (2677 G > T), rs1045642 (3435 C > T), and rs4148738 (intronic in the promoter, A > G) [13]. The first three SNPs are in partial linkage disequilibrium and form several haplotypes [30,31] (Table 3). The rs1045642 and rs4148738 are also in partial linkage disequilibrium [16]. These polymorphisms impact the pharmacokinetics of many P-glycoprotein substrate drugs, but the genotype/phenotype relationship of these variants is not clearly established [32]; only rs1045642 and rs4148738 are associated with increased peak concentration of dabigatran [14,17] (Table 2). In the systematic review and meta-analysis of Xie et al. in 2018, which included a total of 13 clinical studies involving 3144 patients, DOAC peak concentrations in wild homozygous carriers for rs1045642 and rs2032582 of *ABCB1* were lower than those of homozygous mutant carriers; the DOAC peak was also lower in wild homozygous carriers for rs1045642 [25]. However, rs4148738 did not show any impact on the pharmacokinetics of dabigatran [25].

A study on the stability of P-glycoprotein mRNA (messenger ribonucleic acid) by Wang et al. showed an association between the presence of the 3435C > T mutation (rs1045642) and the amount of mRNA present in vitro in human liver samples [33]. Indeed, the substitution of cytosine (C) by thymine (T) would modify the secondary structure of the mRNA by a cis-regulatory mechanism, affecting its stability and thus its quantity in the liver. The two other SNPs, rs1128503 and rs2032582, also induced a secondary structure of mRNA in the model. On the other hand, during in vitro and in vivo experiments, only the 3435C > T mutation was associated with a decrease in P-glycoprotein expression and activity.

##### Epigenetics of *ABCB1*

The synthesis of mRNA, coding for the P-glycoprotein, is synergistically regulated by the genetic variations mentioned above, and epigenetic variations via methylation of the promoter in *ABCB1* gene [34]. Thus, homozygous patients mutated for the haplotype rs1128503-rs2032582-rs1045642 and who have a high methylation rate have the lowest amount of ABCB1 mRNA compared to homozygous mutated patients with a low methylation rate, then to wild homozygous with a high methylation rate, and finally to wild homozygous with a low methylation rate [34].

##### Genetic Polymorphism of *UGT1A9*, *2B7*, and *2B15*

The impact of *UGT1A9*, *2B7*, and *2B15* polymorphisms on systemic exposure to dabigatran has not been studied to date. However, we can assume that their role is likely to be minimal, since they are involved in the production of active metabolites, and in a small proportion [9].

### 3.2. Rivaroxaban

#### 3.2.1. Pharmacodynamics and Pharmacokinetics

Rivaroxaban has an oral bioavailability of approximately 80% [2]. The systemic exposure is increased when rivaroxaban is administered during a meal [35]. Peak plasma concentrations occur 2–4 h after administration. The inter-individual variability of exposure is between 30 and 40%. Rivaroxaban is transported by P-glycoprotein and the breast cancer resistance protein (BCRP) encoded by the *ABCG2* gene [36]. It is highly bound to plasma proteins, in the order of 95% [37]. Two-thirds of the administered dose is metabolized, mainly by cytochrome P450 isoforms 3A4, 3A5, and 2J2, and also by mechanisms independent of CYP450. This metabolization leads to the formation of 18 different inactive metabolites, which are in turn eliminated in the urine (50%) and feces (50%). The remaining third of rivaroxaban is eliminated unchanged in the urine. The mean plasma elimination half-life is 10 h. Rivaroxaban does not induce or inhibit CYP450 [4]. Administration of potent CYP3A4/5 and P-glycoprotein enzyme inhibitors (such as ritonavir, ketoconazole, itraconazole, voriconazole, posaconazole, etc.) increases rivaroxaban plasma concentrations by an average of 2.6-fold, significantly increasing its pharmacodynamics and the risk of bleeding [4]. However, a smaller increase in plasma concentration with other potent CYP3A4/5 and/or P-glycoprotein inhibitors (such as erythromycin, clarithromycin, and fluconazole) was not considered clinically relevant; data for dronedarone are limited [4]. Coadministration of rivaroxaban with potent CYP3A and P-glycoprotein enzyme inducers (rifampin, phenytoin, carbamazepine, phenobarbital, or St. John’s wort) may reduce its plasma concentration [4].

#### 3.2.2. Pharmacogenetics

Concerning the *ABCB1* gene, Ing Lorenzini’s team reported in 2016 a case of rivaroxaban-induced hemorrhage in a patient with homozygous mutated TT genotype for rs2032582 and rs1045642 [20], which is in line with the results highlighted by Xie et al. in 2018 (higher peak concentrations for these homozygous mutated genotypes, as well as AUC for rs1045642) [25]. In Gouin-Thibault’s 2017 study, these two variants did not show a significant increase in rivaroxaban peak concentrations in a cohort of healthy volunteers [16]. Among three patients who experienced major bleeding associated with a residual blood concentration > 136 ng/mL in the 2018 Sennesael study, all were heterozygous for rs1128503, rs2032582, and rs4148738; two were heterozygous and one was a homozygous mutated TT for *ABCB1* rs1045642 [18]. These results are shown in Table 2.

For *CYP3A4*, it was shown in a study by Sychev et al. in 2018 that the peak and trough rivaroxaban concentrations depended on *CYP3A4* activity [38]. In addition, a number of *CYP3A4* polymorphisms are known to decrease its activity, such as CYP3A4*22/rs35599367 [13,39] or CYP3A4*17/rs4987161 [40]. In 2019, another study by Sychev et al. in 78 patients showed no significant difference in peak concentration between the mutated haplotypes *ABCB1*-rs1045642/*CYP3A4*-rs35599367 and *ABCB1*-rs4148738/*CYP3A4*-rs35599367 compared to the respective wild haplotypes [22]. These results are shown in Table 2.

### 3.3. Apixaban

#### 3.3.1. Pharmacodynamics and Pharmacokinetics

Apixaban has an oral bioavailability of approximately 50% [2]. The peak plasma concentration is reached 3–4 h after administration. Intra-individual and inter-individual variabilities are approximately 20% and 30%, respectively [5]. Apixaban is transported by P-glycoprotein and BCRP. Plasma protein binding is high (87%) [37]. A quarter of the absorbed amount is converted to inactive metabolites, mainly by CYP3A4 and CYP3A5, but also by CYP1A2, CYP2C8, CYP2C9, CYP2C19, and CYP2J2 [36], and the sulfotransferases SULT1A1 and SULT1A2 (leading to O-desmethyl-apixaban sulfate), mainly SULT1A1 [41]. A proportion of 27% of apixaban is excreted in urine in an unchanged form. The remaining part of apixaban and inactive metabolites are excreted in the feces. The half-life of apixaban is approximately 12 h [5]. Concomitant administration of potent enzyme inhibitors of CYP3A4/5 and P-glycoprotein increases the blood concentration of apixaban by an average of two-fold [5]. Other active substances, weaker CYP3A4/5 and P-glycoprotein inhibitors (diltiazem, naproxen, clarithromycin, amiodarone, verapamil, quinidine), may increase apixaban plasma concentrations to a lesser extent [5]. Conversely, co-administration of apixaban with CYP3A4/5 and P-glycoprotein enzyme inducers (rifampicin, phenytoin, carbamazepine, phenobarbital, or St. John’s wort) may reduce its plasma concentration [5].

#### 3.3.2. Pharmacogenetics

In 2016, Dimatteo’s team demonstrated an association between the intronic variant rs4148738 of *ABCB1* and an increase in the peak concentration of apixaban (*p* < 0.05) [26]. In 2017, Ueshima’s study of a cohort of 44 Japanese patients treated for non-valvular atrial fibrillation showed a significant increase in the ratio of residual concentration/dose of apixaban with CYP3A5*1/*3 or *3/*3 (rs776746) and *ABCG2* 421A > A (rs2231142) genotypes [13] compared to CYP3A5*1/*1 and *ABCG2* 421C > C genotypes respectively; variants 1236C > T (rs1128503), 2677G > T (rs2032582), and 3435C > T (rs1045642) of the *ABCB1* gene had no impact on this ratio [19]. The 2018 Kruykov study in a sample of 17 Russian patients treated with apixaban, 10 mg daily, did not show a significant impact of *ABCB1* rs1045642 and rs4148738 or *CYP3A5* rs776746 on the pharmacokinetics of apixaban [23]. In 2019, Huppertz reported the case of one woman with dramatically increased apixaban plasma concentrations 3 h (peak) and 12 h after an oral dose: 1100 ng/mL and 900 ng/mL, respectively, compared to the range expected (91 to 321 ng/mL at peak and 41 to 231 ng/mL after 12 h). Four polymorphisms may have result in such increase: *ABCB1* rs2032582, rs1045642, and *CYP3A5* rs776746 were found mutated homozygous, and *ABCG2* rs2231142 was found heterozygous [21]. She also suffered from moderate renal impairment, which could also lead to increased plasma concentrations. Finally, in 2020, Gulilat’s study of 358 Caucasian patients with atrial fibrillation demonstrated the relationship between the *ABCG2* 421C > A variant (resulting in impaired transporter function) and higher peak and trough blood levels of apixaban [27]. These results are shown in Table 2.

The sulfotransferase SULT1A1 has three main allelic variants: SULT1A1*1 (wild type), SULT1A1*2 (638 G > A), and SULT1A1*3 (667 A > G). The effect on apixaban metabolism is very small for SULT1A1*2 and moderate for SULT1A1*3, which could lead to variations in the efficacy of apixaban by variation in its metabolites [41]. To date, no studies have investigated the impact of these variants on the efficacy or toxicity of apixaban.

### 3.4. Edoxaban

#### 3.4.1. Pharmacodynamics and Pharmacokinetics

The bioavailability of edoxaban is around 60% [2]. Absorption is not altered in the presence of food [35]. The peak concentration is reached within 1–2 h. Edoxaban is a substrate for P-glycoprotein. It is 55% bound to plasma proteins [37], and is metabolized by CES1 and CYP3A4/5 to three active metabolites in a small proportion (about 10%), of which M4 is a substrate of the OATP1B1 (organic anion transporter protein 1B1) transporter encoded by the *SLCO1B1* (solute carrier organic anion transporter family, member 1B1) gene [36]. The urinary excretion of edoxaban is 35% remaining of the unchanged fraction, and metabolites are excreted in the feces. Its half-life is 10–14 h [6]. Potent enzyme inhibitors of P-glycoprotein increase systemic exposure to edoxaban by a factor of 1.5 to 2 [6].

#### 3.4.2. Pharmacogenetics

Edoxaban is metabolized mainly by CES1, but very little by CYP3A4/3A5, and is transported by P-glycoprotein. Variations in systemic exposure could be related to the *CES1* and *ABCB1* polymorphisms [42]. To date, only one study has investigated the rs1045642 (3435 C > T) variants of *ABCB1* and rs4149056 (521 T > C) of *SLCO1B1* [13]. These variants do not seem to impact the pharmacokinetics of edoxaban [24] (Table 2).

### 3.5. Betrixaban

#### 3.5.1. Pharmacodynamics and Pharmacokinetics

Betrixaban has an oral bioavailability of approximately 34%. The peak plasma concentration appears within 3–4 h after administration [43,44]. The mean plasma elimination half-life is 20 h, with a terminal half-life of 37 h. Administration with food is recommended to reduce plasma concentration variability [43]. Plasma protein binding is 60% [43,44]. Betrixaban is transported by P-glycoprotein [43], and concomitant use of P-glycoprotein inhibitors results in a 2.5- to five-fold increase in plasma peak concentrations, and a two- to three-fold increase in AUC, depending on the inhibitors [44]. Betrixaban is transformed into two inactive major metabolites by a CYP-independent hydrolysis [44]. Unlike the other factor Xa inhibitors, betrixaban has a minimal (less than 1%) hepatic metabolism by CYP450 (CYP1A1, 1A2, 2B6, 2C9, 2C19, 2D6, and 3A4), which reduces drug–drug interactions [44]. The active drug is excreted unchanged through the biliary system, then the feces for 85% and in urine for 8 to 11% [43,44].

#### 3.5.2. Pharmacogenetics

To date, there is no data on genetic polymorphisms and betrixaban pharmacokinetics and pharmacodynamics. However, one would expect that *ABCB1* polymorphisms could impact plasma concentrations of betrixaban.

### 3.6. Plasma Concentrations and Adverse Events

To the best of our knowledge and to date, there is little data about the relationship between DOAC’s pharmacokinetics and pharmacodynamics. However, two studies are of interest about dabigatran and edoxaban.

Regarding the risk of major bleeding in patients on dabigatran therapy, Reilly previously showed that this risk increased with dabigatran exposure (*p* < 0.0001) [45]. The median trough concentration and post-dose concentration were, respectively, 55% (116 versus 75.3 ng/mL) and 36% higher in patients with major bleeding compared to those without bleeding. Age was also an important covariate (*p* < 0.0001) [45]. No difference was shown in the median plasma concentration between patients with ischemic stroke or systemic embolism and patients who did not experience these events [45].

Ruff et al., based on ENGAGE AF-TIMI 48 trial data, have described the dose–concentration relationship and impact on anti-FXa activity for edoxaban [46]. The reduction from an oral dose of 60 mg to 30 mg and from 30 mg to 15 mg decreased mean exposure by 29% (34.6 versus 48.5 ng/mL) and 35% (16 versus 24.5 ng/mL), respectively, as well as mean anti-FXa activity by 25% and 20%, respectively [46]. Regarding the link between plasma concentrations and adverse events, this trial showed that with increasing edoxaban concentration, a gradual linear decrease in the risk of stroke or systemic embolic events occurred by contrast with the steeper increase in the risk of major bleeding [46]. Overall, the risk of major bleeding exceeded the risk of stroke or systemic embolic events, and the therapeutic window for edoxaban appeared narrower for major bleeding than thromboembolism [46]. Globally, the risk of major bleeding seems to be correlated with increasing plasma levels of direct oral anticoagulants. The risk of stroke or systemic embolic events fluctuates less with concentration variation.

## 4. Discussion and Conclusions: Implementation in Clinical Practice Guidelines

To date, there is no recommendation with a high level of evidence regarding the search for polymorphisms of the *CES1*, *ABCB1*, *CYP3A4*, *CYP3A5*, and *ABCG2* genes as part of therapeutic optimization for patients undergoing DOAC treatment. The methodological evaluation of studies of the association between genetic polymorphisms and cardiovascular drugs using the AGREE (Appraisal of Guidelines, Research, and Evaluation) method demonstrated the good methodological quality of the search for rs2244613 *CES1* polymorphism in patients treated with dabigatran in the same way as the search for *CYP2C19* and clopidogrel, or *CYP2C9* and warfarin polymorphisms. This finding supported the use in clinical practice of this polymorphism of interest in dabigatran-treated patients [47]. In addition, according to the PharmGKB database (www.pharmgkb.org), the search for rs2244613 and rs8192935 *CES1* polymorphisms is indicated at Evidence Level 3 (low) for dabigatran, and for the rs776746 *CYP3A5* and rs2231142 *ABCG2* polymorphisms for apixaban. However, this level of evidence is insufficient to allow implementation of pharmacogenetic testing in clinical practice. This low level of evidence is due to the lack of reproducibility of results between studies [48]. The DAPHNE clinical study involving a cohort of 350 patients on rivaroxaban and apixaban is currently being conducted by Victoria Rollason’s team (University Hospitals of Geneva); it aims to analyze the impact of certain polymorphisms of the *CYP3A4*, *CYP3A5*, *CYP3A7,* and *ABCB1* genes, as well as the phenotyping of the proteins encoded by these genes on the pharmacokinetics of these two DOACs [49]. The results of this trial will be useful to clarify the use of pharmacogenetic testing during DOAC treatment. Randomized controlled trials, similar to those undertaken for *CYP2C9* and *VKORC1* genotyping prior to anti-vitamin K treatment [50,51] or *CYP2C19* genotyping prior to clopidogrel treatment [52], will demonstrate the clinical utility of a priori genotyping of patients before introduction of direct oral anticoagulants [53,54]. In clinical practice, pharmacogenetic testing could help prescribers in choosing the most appropriate DOAC treatment according to each patient’s characteristics with the lowest risk of plasma concentration variability, thus optimizing an individual patient’s risk of bleeding and thromboembolic events. Therapeutic drug monitoring (TDM) could then be use as a complement to individualize oral doses in order to obtain optimal plasma levels. Lastly, there is no clear evidence between hemorrhage risk increase and a particular genetic polymorphism.

## Figures and Tables

**Table 1:** Genes coding for proteins involved in the activation, transport, and metabolism of DOACs [2,3,4,5,6].

DCI	Activation	Transport	Metabolism
Dabigatran	*CES1*, *CES2*	*ABCB1*	*UGT1A9, UGT2B7*, *UGT2B15*
Rivaroxaban	*-*	*ABCB1*, *ABCG2*	*CYP3A4/5*, *CYP2J2*
Apixaban	*-*	*ABCB1*, *ABCG2*	*CYP3A4/5*, *CYP1A2, CYP2J2*
Edoxaban	*-*	*ABCB1*, *SLCO1B1*	*CES1*, *CYP3A4/5*
Betrixaban		*ABCB1*	*CYP450-independent hydolysis*

*ABCB1*: ATP-binding cassette isoforme B1; *ABCG2*: ATP-binding cassette isoforme G2; *CES*: carboxyesterase; *CYP*: Cytochrome P450; *SLCO1B1*: solute carrier organic anion transporter family, member 1B1; *UGT*: UDP-glucuronyltransferase.

**Table 2:** Pharmacokinetic variations in DOACs based on genetic polymorphisms of *CES1*, *ABCB1*, *CYP3A4*, *CYP3A5*, *ABCG2*, and *SLCO1B1*.

Gene SNP Allelic Change Amino Acid Change Frequency	*DABIGATRAN*	*RIVAROXABAN*	*APIXABAN*	*EDOXABAN*	*BETRIXABAN*
***CES1*** **rs2244613** intron: C > A - C = 0.266 [13]	↓ [trough] by 15% per mutated allele (*p* = 1.2 × 10^−8^) [14] ↓ risk of bleeding (*p* = 7 × 10^−5^) [14] ↓ bleeding compared to warfarin for mutated alleles (*p* = 0.002) [14] Not associated with ischemic events [14] ↓ [trough] of dabigatran (*p* = 0.04) HTZ = 2% and MT = 3% [15] No effect on AUC (NS) or [peak] (NS) [16] ↓ [trough] for mutated alleles carriers (NS) [17]	*NI*	*NI*	*NI*	*NI*
***CES1*** **rs8192935** intron: T > C - T = 0.420 [13]	↓ [peak] by 12% (*p* = 3.2 × 10^−8^) [14] Not associated with ischemic or bleeding events [14] ↓ [trough] (*p* = 0.033) HTZ = 3% and MT «TT» = 11% [15]	*NI*	*NI*	*NI*	*NI*
***CES1*** **rs71647871** 536 G > A 143 Gly > Glu A = 0.014 [13]	Loss of CES1 function: ↓ by 41% of the transformation of the prodrug and metabolites in dabigatran (*p* = 0.026 for BIBR 951) [12]	*NI*	*NI*	*NI*	*NI*
***ABCB1*** **rs1128503** 1236 C > T 412 Gly > Gly T = 0.46 [13]	Results not significant for AUC and [peak] of dabigatran Haplotype HTZ: *p* = 0.61 Haplotype MT: *p* = 0.58 [16]	Major bleeding under rivaroxaban for three MT patients [18]	No impact on [trough]/dose ratio for apixaban [19]	*NI*	*NI*
***ABCB1*** **rs2032582** 2677 G > T/A 893 Ala > Ser/Thr T = 0.42 A = 0.08 [13]	Results not significant for AUC and [peak] of dabigatran Haplotype HTZ: *p* = 0.61 Haplotype MT: *p* = 0.58 [16]	One case of rivaroxaban-induced hemorrhage with homozygous mutated genotypes ‘TT’ [20] No significant increase of rivaroxaban [peak] [16] Major bleeding under rivaroxaban for three MT patients [18]	No impact on [trough]/dose ratio for apixaban [19] One case of highly increased [peak] and concentration 12 h post dose in a homozygous patient (TT), along with other mutations on *ABCB1* (rs1045642, MT), *ABCG2* (rs2231142, HTZ), and *CYP3A5* (rs776746, MT) [21]	*NI*	*NI*
***ABCB1*** **rs1045642** 3435 C > T 1145 Ile > Ile T = 0.50 [13]	Results not significant for AUC and [peak] of dabigatran Haplotype HTZ: *p* *=* 0.61 Haplotype MT: *p* *=* 0.58 [16] Associated with ↑ [peak] of dabigatran and ↑ risk of bleeding complications (*p* < 0.008) [17]	One case of rivaroxaban-induced hemorrhage with homozygous mutated genotypes ‘TT’ [20] No significant increase of rivaroxaban [peak] [16] Major bleeding under rivaroxaban for three MT patients [18] No significant increase of rivaroxaban [peak] in mutated patients compared to wild type (haplotype of *ABCB1* rs1045642 and *CYP3A4* rs35599367) [22]	No impact on [trough]/dose ratio for apixaban [19] No impact on apixaban pharmacokinetics [23] One case of highly increased [peak] and concentration 12 h post dose in a homozygous patient (TT), along with other mutations on *ABCB1* (rs2032582, MT), *ABCG2* (rs2231142, HTZ), and *CYP3A5* (rs776746, MT) [21]	It seems to have no impact on the pharmacokinetics of edoxaban [24]	*NI*
***ABCB1*** **rs4148738** intron: A > G - G = 0.38 [13]	Associated with ↑ [peak] by 12% (*p* = 8.2 × 10^−8^), but not associated with ischemic or bleeding events [14] No effect on [trough] and [peak] of dabigatran [15] Associated with ↑ [peak] of dabigatran [17] No impact on dabigatran pharmacokinetics [25]	Major bleeding under rivaroxaban for three MT patients [18]	Associated with ↑ [peak] of apixaban (*p* = 0.048) [26] No impact on apixaban pharmacokinetics [23]	*NI*	*NI*
***CYP3A4*** **rs35599367** intron: C > T - T = 0.03 [13]	*NI*	No significant increase of rivaroxaban [peak] in mutated patients compared to wild type (haplotype of ABCB1 rs1045642 and CYP3A4 rs35599367) [22]	*NI*	*NI*	*NI*
***CYP3A5*** **rs776746** intron: T > C - T = 0.29 [13]	*NI*	*NI*	Significant ↑ of ratio [trough]/dose of apixaban in HTZ or MT patients [19] One case of highly increased [peak] and concentration 12 h post dose in a MT patient, along with other mutations on *ABCB1* (rs2032582 and rs1045642, MT), and *ABCG2* (rs2231142, HTZ) [21] No impact on apixaban pharmacokinetics [23]	*NI*	*NI*
***ABCG2*** **rs2231142** 421 C > A 141 Gln > Lys A = 0.12 [13]	*NI*	*NI*	Significant ↑ of [trough]/dose ratio of apixaban in MT patients [19] One case of highly increased [peak] and concentration 12 h post dose in an HTZ patient, along with other mutations on *ABCB1* (rs2032582 and rs1045642, MT), and *CYP3A5* (rs776746, MT) [21] ↑ [peak] et [trough] of apixaban [27]	*NI*	*NI*
***SLCO1B1*** **rs4149056** 521 T > C 174 Val > Ala C = 0.13 [13]	*NI*	*NI*	*NI*	It seems to have no impact on the pharmacokinetics of edoxaban [24]	*NI*

AUC: area under curve; MT: mutated homozygous; HTZ: heterozygous; ↓: decrease; ↑: increase; [peak]: peak concentration; [trough]: trough concentration; NI: no information; NS: non significant.

**Table 3:** *ABCB1* haplotypes.

*ABCB1* SNP	rs1128503	rs2032582	rs1045642	rs10276036 Intronic	rs2235033 Intronic	rs2235013 Intronic
ABCB1*1 (Kim et al.) [30]	C	G	C	G	T	G
ABCB1*2 (Kim et al.) [30]	T	T	T			
ABCB1*2 (Kroetz et al.) [31]	C	G	T	G	T	G
ABCB1*13 (Kroetz et al.) [31]	T	T	T	A	C	A

A: adenine; C: cytosine; G: guanine; T: thymine. Several definitions of the haplotype have been made according to the teams. The haplotypes ABCB1*2 of Kim et al. and ABCB1*13 of Kroetz et al. can be differentiated by three intronic SNPs (rs10276036, rs2235033, and rs2235013).

## Data Availability

Data sharing is not applicable to this article.
